# Reconstruction and analysis of genome-scale metabolic model of a photosynthetic bacterium

**DOI:** 10.1186/1752-0509-4-156

**Published:** 2010-11-17

**Authors:** Arnau Montagud, Emilio Navarro, Pedro Fernández de Córdoba, Javier F Urchueguía, Kiran Raosaheb Patil

**Affiliations:** 1 Instituto Universitario de Matemática Pura y Aplicada, Universidad Politécnica de Valencia, Camino de Vera 14, 46022 Valencia, Spain; 2 Departamento de Lenguajes y Ciencias de la Computación, Campus de Teatrinos, Universidad de Málaga, 29071 Málaga, Spain; 3 Structural and Computational Biology Unit, European Molecular Biology Laboratory, Meyerhofstrasse 1, D-69117 Heidelberg, Germany

## Abstract

**Background:**

*Synechocystis *sp. PCC6803 is a cyanobacterium considered as a candidate photo-biological production platform - an attractive cell factory capable of using CO_2 _and light as carbon and energy source, respectively. In order to enable efficient use of metabolic potential of *Synechocystis *sp. PCC6803, it is of importance to develop tools for uncovering stoichiometric and regulatory principles in the *Synechocystis *metabolic network.

**Results:**

We report the most comprehensive metabolic model of *Synechocystis *sp. PCC6803 available, *i*Syn669, which includes 882 reactions, associated with 669 genes, and 790 metabolites. The model includes a detailed biomass equation which encompasses elementary building blocks that are needed for cell growth, as well as a detailed stoichiometric representation of photosynthesis. We demonstrate applicability of *i*Syn669 for stoichiometric analysis by simulating three physiologically relevant growth conditions of *Synechocystis *sp. PCC6803, and through *in silico *metabolic engineering simulations that allowed identification of a set of gene knock-out candidates towards enhanced succinate production. Gene essentiality and hydrogen production potential have also been assessed. Furthermore, *i*Syn669 was used as a transcriptomic data integration scaffold and thereby we found metabolic hot-spots around which gene regulation is dominant during light-shifting growth regimes.

**Conclusions:**

*i*Syn669 provides a platform for facilitating the development of cyanobacteria as microbial cell factories.

## Background

Cyanobacteria, which have been model organisms since the early 70s of the past century [1], are a widespread group of photoautotrophic microorganisms, which originated, evolved, and diversified early in Earth's history [2]. It is commonly accepted that cyanobacteria played a crucial role in the Precambrian phase by contributing oxygen to the atmosphere [3]. All cyanobacteria combine the ability to perform an oxygenic photosynthesis (resembling that of chloroplasts) with typical prokaryotic features, like performing anoxygenic photosynthesis by using hydrogen sulfide (H_2_S) as the electron donor or fixing atmospheric dinitrogen (N_2_) into ammonia (NH_3_). Relevance of this phylum covers from evolutionary studies [4] to biotechnological applications, including biofuel production [5]. *Synechocystis *sp. PCC6803 is a cyanobacterium that is considered as a good candidate for developing a photo-biological cell factory towards production of a variety of molecules of socio-economic interest, with CO_2 _(and/or sugars) as carbon source and light (and/or sugars) as energy source [6]. The diversity of potential applications in this sense is broad. Works have been published on heterologous production of metabolites such as isoprene [6], poly-beta-hydroxybutyrate [7], biofuels [8] and bio-hydrogen [9,10] - an energy vector of global interest [11].

*Synechocystis *sp. PCC6803 is capable of growing under three different growth conditions as marked by the utilized carbon source (/s) [12]. This causes that three distinct modes of operation are interweaved over the same metabolic network, *viz*., i) photoautotrophy, where energy comes from light and carbon from CO_2_; ii) heterotrophy, where energy and carbon source is a saccharide, for instance glucose; and, iii) mixotrophy, a combination of the above two, where light is present as well as a combination of two carbon sources: glucose and CO_2_. Reconstruction of a genome-scale metabolic model for this model photo-synthetic bacterium is one of the main goals of the current study. Genome-scale metabolic network reconstruction is, in essence, a systematic assembly and organization of all the reactions which build up the metabolism of a given organism; and has been of great interest in the post-genomic era. The variety of applications of such a metabolic model [13] includes the possibility of assessing projects for the production and optimization of an added value metabolite. If a model is formulated properly, it is expected to allow simulating environmental and genetic perturbations in the metabolic network. Thus, together with appropriate constraints, a metabolic model would partially represent a virtual organism - an *in silico *model that allows probing possible flux distributions inside the cell under different environmental conditions and for a given genetic make-up. Towards this end, a variety of tools/algorithms are available [14], including flux balance analysis (FBA) [15,16], minimization of metabolic adjustments (MOMA) [17], regulatory on-off minimization (ROOM) [18] and metabolic control analysis (MCA) [19,20].

*Synechocystis *sp. PCC6803 genome was sequenced, annotated and made publicly available in 1996 [21,22] and has been the target of some metabolic modeling effort, especially for central carbon metabolic reconstructions [23,24]. The work from Yang *et al *[23] focused on a metabolic model of glycolysis, tricarboxylic acid cycle and pentose phosphate pathway that was simulated under heterotrophic and mixotrophic conditions. Shastri and Morgan [24] studied a metabolic model with the same pathways under autotrophic conditions and compared their results to the ones from Yang *et al*. These two works considered one lumped reaction for the photosynthesis of the system. More recently, an uncurated reaction list with a biomass composition represented by central carbon metabolites has been published [25]. This model, however, is not suitable for simulations due to lack of proper biomass equation, lumped nature of some key reactions and missing reactions.

The large quantity of information featured in public databases, like details about genomes [26], pathways [27], enzymes [28] or proteins [29] can be used from different databases to gather all published data for one specific organism. However, the lack of quality must be considered as a major drawback of some of the databases: false positives, false negatives as well as wrongly annotated objects may hinder efforts of collecting accurate data [30]. Consequently, manual reconstruction by detailed inspection of each and every reaction, biomass equation based on metabolic building blocks (such as amino acids and nucleotides), consistency and integrity of the network is a pre-requisite for creating a high quality and useful metabolic model [31]. The current study presents such manually curated reconstruction for *Synechocystis *sp. PCC6803 and demonstrates some of its potential applications.

The present model features a detailed biomass equation which encompasses all the building blocks that are needed for a flux distribution simulation that reflects observed phenotype. No lumped reactions are present and photosynthesis is described as a set of 19 reactions, thus enabling the tracing of the corresponding fluxes. Furthermore, different analyses are performed by using this metabolic reconstruction, including reaction knock-out simulations, flux variability analysis and identification of transcriptional regulatory hotspots. Overall, *i*Syn669 is a valuable tool towards the development of a photo-biological production platform. The model will also contribute to the existing set of genome-scale models with a virtue of being one of the first stoichiometric models that account for photosynthesis.

## Results and Discussion

### Genome-scale metabolic network reconstruction

A complete literature examination, including databases, biochemistry textbooks and the annotated genome sequence, was needed in order to extract the current state of the art on known metabolic reactions within the metabolic network of *Synechocystis *sp. PCC6803. For a thorough overview of the process of metabolic model reconstruction, refer to very instructive work by Forster *et al *[32] as well as review by Feist *et al *[31]. In detail, the reconstruction started with the annotation and genomic sequence files of *Synechocystis *sp. PCC6803 [21,22]. These files were used with Pathway Tools software [33] in order to build a database of all the genes, proteins and metabolites presents in the organism. The list of reactions was then retrieved from Pathway Tools; EC numbers and stoichiometry of the reactions were checked and verified with the help of the Enzyme nomenclature database [34] and KEGG pathway database [27]. Reactions were elementally balanced except for protons, so that chemical conversions were coherent. In some of the reactions present in these databases, metabolites were reported in a non-specific form (e.g. 'an alcohol'). This is insufficient for metabolic model simulation and, so, corresponding organism-specific metabolites had to be identified [32]. Additionally, in a large number of reactions cofactors were not completely clarified: an enzyme being capable of using NADH or NADPH or both. In the latter, two reactions were included in the reconstructed metabolic network. Determination of reversibility of the reactions was assisted by specific enzyme databases, like BRENDA [28]. If no conclusive evidence was reported, reactions were set to be reversible.

In the reconstruction of the metabolic model, many reactions (a total of 79 reactions, see Table 1) were found to be necessary for the production of the monomers, precursors or building blocks, that are considered in the biomass equation but which have no corresponding enzyme coding gene assigned. In consequence, many genes that were not annotated before should be considered, as they code for enzymes that should be present to allow the formation of biomass. For instance, enzymes malyl-CoA lyase and isocitrate lyase were not allocated in the annotation of the genome albeit their activities have been measured [35,36] and their presence is necessary to complete the glyoxylate shunt; consequently, they were included in the model.

**Table 1:** Distribution of the model reactions as per cognate genes

Number of reactions	882
-With assigned genes	669

·Protein-mediated transport	78

-With no cognate gene	221

·Chemical conversion	47

·Transport reactions	20

·EC reactions not annotated	79

·Needed for biomass simulation	75

The product of this reconstruction process was a set of reactions that encompass all the known metabolite conversions that take place in *Synechocystis *sp. PCC6803. The resulting network, *i*Syn669, consists of 882 metabolic reactions and 790 metabolites (see Table 1 for more information). A total of 669 genes were included, to which 639 reactions were assigned (see Additional file 1 for details); the difference between the number of genes and assigned reactions is due to the presence of considerable number of protein complexes (e.g. photosynthetic or respiratory activities) and isoenzymes. Reactions with no cognate genes are also present in *i*Syn669, 20 passive transport reactions and 47 chemical conversions (not mediated by enzymes) were included. Additionally, a total of 79 reactions were included on the basis of biochemical evidence or physiological considerations, but currently with no annotated Open Reading Frame (ORF). *i*Syn669 genome-scale metabolic model is available in Additional file 2 (in OptGene [37] format).

*i*Syn669 spans all the biologically relevant flux nodes in the *Synechocystis *metabolism. Pyruvate, phosphoenolpyruvate (PEP), 3-phosphoglycerate, erythrose-4-phosphate and 2-oxoglutarate are main flux nodes for amino acids biosynthesis. Acetyl-CoA is an important flux node for fatty acids production, with high relevance for metabolic engineering towards biofuel production. Biosynthesis of nucleic acids comes from different metabolites, namely, ribose-5-phosphate, 5-phospho-beta-D-ribosyl-amine, L-histidine and L-glutamine. Moreover, with the information publicly available on databases, we can conclude that *Synechocystis *sp. PCC6803 bears an incomplete tricarboxylic acid cycle (TCA cycle), as it lacks 2-ketoglutarate dehydrogenase (EC 1.2.4.2). It has been published that glyoxylate shunt completes this cycle [35], permitting the recycling of TCA metabolites. Alternatively, aspartate transaminase (reaction *2.6.1.1a *in *i*Syn669) can interconvert 2-ketoglutarate and oxaloacetate, thus bridging the gap of 2-ketoglutarate dehydrogenase, but short-circuiting TCA cycle.

From the network topology perspective, *i*Syn669 displays the connectivity distribution pattern similar to that of the other microbial genome-scale networks, e.g. yeast [32] and *Escherichia coli *[38] (Table 2). While most of the metabolites have few connections, few metabolites are involved in very many reactions and are often referred to as metabolic hubs. Homeostasis of such highly connected metabolites will affect globally the metabolic phenotype (as reflected in metabolite levels and fluxes) and therefore of interest for studying the organization of regulatory mechanisms on the genome-wide scale. Most connected metabolites include those related to energy harvesting (e.g. ATP, NADP+, oxygen), a key metabolite in the porphyrin and chlorophyll metabolism (S-adenosyl methionine), a couple of amino acids and its precursors (L-glutamate, L-glutamine and glutathione) and a key metabolite in the lipid biosynthesis pathway (malonyl-ACP). High connectivity of these metabolites hints to their potential central role in the re/adjustments of fluxes following environmental changes/perturbations. In order to discover the corresponding regulatory mechanisms, additional studies should be done - e.g. putative regulatory sequence motifs associated with the neighbors of these highly connected metabolites [39]. Furthermore, most connected metabolites with filtered cofactors can be found in Additional file 3.

**Table 2:** Most connected metabolites in the iSyn669 metabolic network.

*Metabolite*	*Neighbors*	*Neighbors* *in *E. coli	*Neighbors* *in yeast*
H2O	213	697	-

ATP	144	338	166

phosphate	108	81	113

ADP	103	253	131

diphosphate	97	28	-

H+	74	923	188

CO2	72	53	66

NADP+	64	39	61

NADPH	63	66	57

NAD+	46	79	58

L-glutamate	45	52	56

NADH	42	75	52

AMP	36	86	48

oxygen O2	36	40	31

ammonia	28	22	-

S-adenosyl-L-methionine	25	18	19

glutathione	25	17	10

a malonyl-ACP	23	15	10

L-glutamine	22	18	23

coenzyme A	21	71	39

### Simulations of the three metabolic modes

*i*Syn669, together with appropriate physiological constraints, was used as a stoichiometric simulation model by using FBA algorithm [40]. The FBA model simulates steady state behavior by enforcing mass balances constraints for the all metabolic intermediates (Methods). Biomass synthesis, a theoretical abstraction for cellular growth, is considered as a drain of some of these intermediates, i.e. building blocks, into a general biomass component. Different studies have reported that the simulation results do not usually vary drastically when using a common biomass equation for different growth condition [15,24]. Nevertheless, experimental efforts should be directed at the depiction of the best precursors and composition that could characterize, at least, the three main growth modes, *viz*., autotrophy, heterotrophy and mixotrophy, in the scope of recent results [41]. Due to the lack of such data, the present work uses one single biomass equation in the simulations of all three metabolic states (Table 3). Presence of photosynthesis allows *i*Syn669 to "grow" under the all three metabolic states (*i.e*., FBA with biomass formation as an objective function results in a feasible solution): carbon dioxide and light (autotrophic), sugars (heterotrophic), carbon dioxide, light and sugars (mixotrophic).

**Table 3:** iSyn669 Biomass composition.

*Metabolite*	*mmole/g DCW*	*Metabolite*	*mmole/g DCW*
**Amino acids **[38]		**Deoxyribonucleotides **[58]	

Alanine	0.499149	dATP	0.0241506

Arginine	0.28742	dTTP	0.0241506

Aspartate	0.234232	dGTP	0.02172983

Asparagine	0.234232	dCTP	0.02172983

Cysteine	0.088988	**Ribonucleotides **[1]	

Glutamine	0.255712	AMP	0.14038929

Glutamate	0.255712	UMP	0.14038929

Glycine	0.595297	GMP	0.12374585

Histidine	0.092056	CMP	0.12374585

Isoleucine	0.282306	**Lipids **[59]	

Leucine	0.437778	16C-lipid	0.20683718

Lysine	0.333448	(9Z)16C-lipid	0.01573412

Methionine	0.149336	18C-lipid	0.00351776

Phenylalanine	0.180021	(9Z)18C-lipid	0.03188596

Proline	0.214798	(9Z,12Z)18C-lipid	0.03568367

Serine	0.209684	(9Z,12Z,15Z)18C-lipid	0.01797109

Threonine	0.246506	(6Z,9Z,12Z)18C-lipid	0.05031906

Tryptophan	0.055234	(6Z,9Z,12Z,15Z)18C-lipid	0.01448179

Tyrosine	0.133993	**Antenna chromophores **[60]	

Valine	0.411184	Chlorophyll a	0.02728183
**Carbohydrates **[61]		Carotenoids	0.00820225

Glycogen	0.01450617		

Biomass composition description with references where the information was retrieved from. All this building blocks with their respective stoichiometric coefficient is converted into one gram of dry cell weight. Biomass equation is reaction *Biomass *in Additional files 2 and 4.

Growth under pure heterotrophy, or dark heterotrophy (in the absence of light) is a subject under study [42,43], being the regular experimental design to give a short light pulse prior to the pure heterotrophic phase (light-activated heterotrophy). Nevertheless, the theoretical flux distribution under heterotrophic conditions is interesting by itself - especially in comparison with the flux distribution in a light-fed energy metabolism. Moreover, fluxes in the heterotrophy mode may help in obtaining insight into the variations under the mixotrophic condition, which is of high relevance for industrial applications [9].

**Figure 1:**
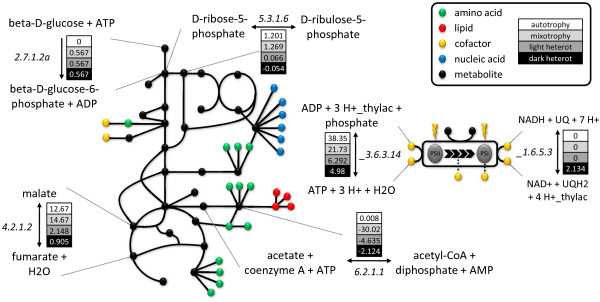
**Selected reactions in *i*Syn669 network that display flux changes across the four studied growth modes**. Flux values (in mmol g_DW_^-1 ^h^-1^) for selected reactions in the *Synechocystis *sp. PCC6803 metabolism. These reactions mark changes across four growth modes, *viz*., autotrophy, mixotrophy and light and dark heterotrophy. Corresponding flux ranges can be found in Table 4 and in Additional file 4 for all the reactions in *i*Syn669.

All FBA simulations were carried out under the appropriate constraints so as to match an autotrophic specific growth rate of 0.09 h^-1^. This growth rate corresponds to a light input of 0.8 mE g_DW_^-1 ^h^-1 ^and to a net carbon flux of 3.4 mmol g_DW_^-1 ^h^-1 ^into the cell, with HCO_3_^- ^and CO_2 _as carbon sources. For the sake of comparison across the different conditions, uptake rates for the corresponding carbon sources were matched based on normalization per number of carbon atoms (this does not affect mono-carbon compounds like carbon dioxide and carbonic acid, but has importance in glucose feeding). Results of the subsequent FBA simulations for the three different growth conditions are presented in the following. Some of the reactions that are physiologically relevant for each of the conditions are summarized in Table 4 and Figure 1. Flux values for the rest of the reactions, including the upper and lower bounds are provided in Additional file 4.

**Table 4:** Comparison of selected fluxes across different growth conditions.

*Reaction* *name*	*Autotrophy*	*Minimum flux*	*Maximum flux*	*Mixotrophy*	*Minimum flux*	*Maximum flux*	*Dark Heterotrophy*	*Minimum flux*	*Maximum flux*	*Light Heterotrophy*	*Minimum flux*	*Maximum flux*	*Reaction description*
*2.7.1.2a*	0	0	0	0.567	0.566	0.567	0.567	0.566	0.567	0.567	0.566	0.567	beta-D-glucose + ATP → beta-D-glucose-6-phosphate + ADP

*4.2.1.2*	12.67	12.667	+∞	14.67	14.657	+∞	0.905	0.884	+∞	2.148	1.836	+∞	malate ↔ fumarate + H2O

*5.3.1.6*	1.201	1.2	+∞	1.269	1.269	+∞	-0.054	-0.051	-0.055	0.066	0.067	+∞	D-ribose-5-phosphate ↔ D-ribulose-5-phosphate

*_UQ*	0.8	0	0.8	0.8	0	0.8	0	0	0	0.8	0	0.8	PSII* + UQ + 2 H+ → PSII + UQH2

*_1.6.5.3*	0	0	+∞	0	0	+∞	2.134	0	+∞	0	0	+∞	NADH + UQ + 7 H+ → NAD+ + UQH2 + 4 H+_peribac

*_3.6.3.14*	38.348	15.7	+∞	21.727	21.7	+∞	4.98	4.95	+∞	6.292	6.281	+∞	3 H+_peribac + phosphate O4P + ADP ↔ 3 H+ + H2O + ATP

*6.2.1.1*	0.008	-∞	+∞	-30.017	-∞	+∞	-2.124	-∞	+∞	-4.635	-∞	+∞	coenzyme A + acetate + ATP ↔ acetyl-CoA + diphosphate + AMP

Units in mmol g_DW_^-1 ^h^-1^. *2.7.1.2a*, glucokinase, is the reaction that phosphorylates beta-D-glucose upon entrance in the cell, marking the start of the glycolysis. The flux direction changes can be seen in reaction *4.2.1.2*, fumarate hydratase, from TCA cycle and *5.3.1.6*, ribose-5-phosphate isomerase, from the pentose phosphate pathway. *_UQ *and *_1.6.5.3 *are reactions that reduce UQH2 from photosystem II or NADH oxidation, respectively, causing a pumping of protons to the thylakoid. *_3.6.3.14 *is the ATP synthase that forms ATP shuttling protons from the thylakoid to the cytosol. *6.2.1.1*, acetate-CoA ligase, is the reaction that generates acetyl-CoA from acetate and coenzyme A, that would be a major flux hub in an ethanol-producing strain, standing as the first step of fermentation.

#### Heterotrophy

Heterotrophy was simulated by considering glucose as the sole carbon source with uptake rate of 0.567 mmol g_DW_^-1 ^h^-1^, entering the system through *glcP *glucose transporter (reaction *TRANS-RXN59G-152 *in *i*Syn669). With the purpose of having a *pure *heterotrophic state, photon uptake rate was constrained to 0; this caused photosynthesis fluxes to be shut down. In this case, glucose will be the source for the formation of carbon backbones for the building blocks of the cell, depicted in the biomass equation. The glycolytic and the oxidative mode of the pentose phosphate pathway were found to be active. Oxidative pentose phosphate pathway is the major pathway for glucose catabolism as was reported in reference [44]. PEP carboxylase (reaction *4.1.1.31 *in *i*Syn669) is the main anaplerotic flux to the TCA cycle. Carbon fixation efficiency is around 60%, the rest being released in the form of CO_2_, as reported in our previous work [9].

In contrast to dark heterotrophy, if a light-activated heterotrophy simulation is run, light enters the system and RuBisCO enzyme is active (reaction *4.1.1.39*), fixing all the CO_2 _that was released in dark heterotrophy, boosting carbon efficiency to a theoretical 100%. In this case, global flux distribution as well as flux ranges resemble that of autotrophy more than that of the dark heterotrophy. Carbon skeletons are still produced through glycolysis and NAD(P)H is reduced along the glycolysis, pyruvate metabolism and TCA cycle. On the other hand, pentose phosphate pathway has shifted to the reductive mode due to RuBisCO activation and the corresponding flux is increased in magnitude. Carbon fixation happens at the RuBisCO level, thereby assimilating the CO_2 _produced by the glucose metabolism, and the production of ATP and NADPH through photosynthesis relieves the oxidative phosphorylation from draining NADPH to generate ATP.

#### Autotrophy

Photoautotrophy was initially simulated considering an illumination of 0.15 mE m^-2 ^s^-1^. Assuming that the mass of a typical *Synechocystis *sp. PCC6803 cell is 0.5 pg [45] and its radius is 1.75 μm [46], we estimated that the theoretical maximum illumination is 41563.26 mE g_DW_^-1 ^h^-1^. An additional optimization step was performed in order to estimate physiologically meaningful photon uptake values that are closer to the experimental measurements [24]. First, carbon uptake rate was found that resulted in a specific growth rate of 0.09 h^-1^, while the light intake was unconstrained. Next, the growth rate was constrained to this value and the second optimization problem was solved where light uptake was minimized. This minimization resulted in photon uptake for photosystem I (reaction *_lightI*) and photosystem II (reaction *_lightII*) being 0.8 mE g_DW_^-1 ^h^-1^. Carbon sources used in simulating photoautotrophy conditions were carbon dioxide and carbonic acid, and its entrance to the system was mediated by RuBisCO (reaction *4.1.1.39 *in *i*Syn669) and carbonic anhydrase (reaction *4.2.1.1b*) respectively. As *i*Syn669 biomass equation encompasses all essential metabolite precursors, these will be the sinks of our network, while photons, carbon dioxide and/or carbonic acid will be the sources. Thus autotrophic fluxes will flow in the gluconeogenic direction and through the Calvin cycle, which is the reductive mode of the pentose phosphate pathway. PEP carboxylase is the main anaplerotic flux to the TCA cycle and glyoxylate shunt is inactive.

#### Mixotrophy

Photons, carbon dioxide and glucose are independent feed fluxes in this simulation. These fluxes entered the system through the same reactions as described for the previous growth modes. Carbon source presents, in this case, one more degree of freedom than in the rest of the conditions. In order to keep a comparative criterion across conditions, we normalized CO_2 _and glucose inputs to the same carbon uptake flux as in the case of the autotrophy and the heterotrophy. Photon uptake rates were also normalized in a similar manner to match the autotrophic state. Having the same metabolic sinks as the two previous modes and the sources from the both of them, it is logical to think that the resulting flux distribution will be a mixture of the autotrophic and heterotrophic simulations. Indeed, we observed that the mixotrophic flux distribution lies in-between the previous two states, being a bit closer to the heterotrophy. Glycolysis is present and glyoxylate is shut down; an active photosynthesis is present, oxidative phosphorylation is less stressed than in heterotrophy as the energy can be produced from the photon uptake; and Calvin cycle is active, as carbon sources are CO_2 _and glucose.

#### Flux variability analysis

**Figure 2:**
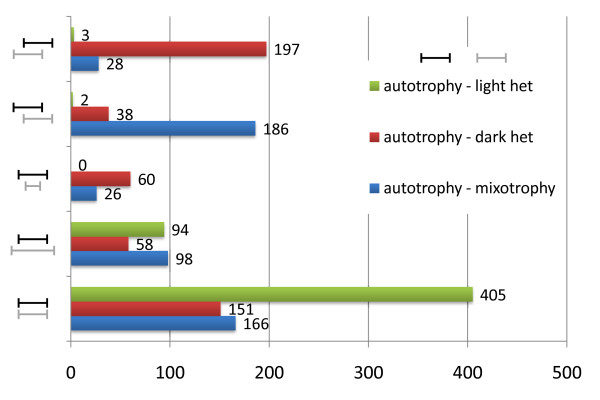
**Overview of the flux adjustments between different growth conditions**. Comparison of flux variability between autotrophy *vs*. mixotrophy, autotrophy *vs*. dark heterotrophy and autotrophy *vs*. light-activated heterotrophy. Minimum and maximum flux ranges were compared for each reaction, 378 reactions were found blocked in all the studied conditions.

Flux balance analysis presented above guarantees to find the optimal objective function value (biomass formation rate). However, the predicted intra-cellular flux distribution is not necessarily unique due to the presence of multiple pathways that are equivalent in terms of their overall stoichiometry. Thus, often the system exhibits multiple optimal solutions and further elucidation requires additional constraints based on experimental evidences (e.g. carbon labeling data). Alternatively, physiological insight can be still obtained by studying the variability at each flux node given the objective function value - a procedure referred to as flux variability analysis. In order to gain insight into the flux changes underlying the changes in the *Synechocystis *metabolism due to (un)availability of light, we have compared the autotrophic growth with the other two by using flux variability analysis (Figure 2). Interestingly, autotrophy permits an overall broader flux landscape than heterotrophy (let it be dark or light-activated). On the other hand autotrophic flux ranges are in general narrower than the mixotrophic ranges. Figure 1 and Table 4 depict some of the physiologically relevant reactions for which the feasible flux range differs across conditions. These include glucokinase from glycolysis, fumarate hydratase from TCA cycle, ribose-5-phosphate isomerase from pentose phosphate pathway, NADH dehydrogenase from oxidative phosphorylation or photosystem II oxidation. These reactions mark the key nodes in the metabolism network that must be appropriately regulated in order to adapt in response to the available energy/carbon source. Mechanisms underlying such changes will be of particular interest not only for biotechnological applications but also from the biological point of view. As a glimpse of the detailed flux (re-)distributions in each of the studied growth conditions, Additional file 5 describes fluxes in the pyruvate metabolism.

### Gene/Reaction knock-out analysis

The comprehensive set of reconstructed biochemical equations of *i*Syn669 and FBA simulations enabled us to further analyze the characteristics and potential of the *Synechocystis *metabolic network. This can be oriented towards the study of the reactions (and thereby the corresponding genes) that are necessary for the growth, or to *in silico *metabolic engineering for identification of targets for maximization of a given metabolite of socio-economic interest.

#### Essential Genes

**Figure 3:**
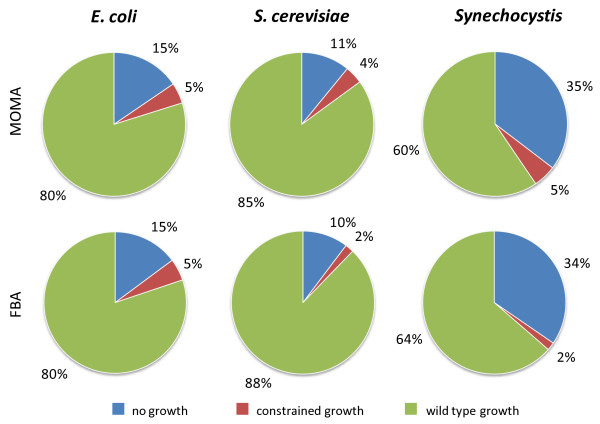
**Essential genes in *Synechocystis *sp. PCC6803**. Distribution of gene knock-out results for three model organisms, simulated by using FBA and MOMA algorithm, classified as wild-type growth, constrained growth and no growth.

*i*Syn669 network consists of 790 metabolites and 882 reactions. Among these, 350 genes (36% of the total, Figure 3) were found to be necessary for the formation of the biomass under the mixotrophic growth conditions by using FBA and MOMA algorithms. This set of genes can be divided in to two categories: i) essential genes, deletion of which completely inhibits biomass growth (304 genes, 34% of the total, with FBA): and ii) genes deletion of which causes a reduced growth rate (46 genes, 2% of the total, with FBA). The set of 304 essential genes can be understood as the core of the metabolism, as deleting them would produce an unviable organism. The results based on MOMA algorithm essentially tally these numbers: 311 essential genes, 35% of the total, and 45 that cause a reduced growth rate, 5% of the total, (Additional file 6).

Interestingly, if we compare the proportion of the essential genes under FBA simulation in the metabolic networks of *E. coli *(187 genes, 15% of the total) [38] and *Saccharomyces cerevisiae *(148, 10% of the total) [32] with *i*Syn669, we find that *Synechocystis *has a significantly larger fraction of metabolic genes whose deletion obliterates biomass formation (304 genes, 34% of the total). One possible explanation for the difference in the relative proportion of essential genes in these three organisms would be an incomplete/incorrect annotation of the genome of *Synechocystis *sp. PCC6803. For example, if only one of the isoenzymes corresponding to a reaction is annotated, the corresponding *in silico *knock-out will result in a false negative prediction. It is important to note that the computational predictions of gene essentiality based on FBA are highly dependent on the growth medium used for the simulations. Thus, the comparison across different species may not be straight-forward. Moreover, it is also possible that the natural growth conditions of *Synechocystis *may have dictated selection for a relatively high proportion of essential genes. Such hypotheses need careful consideration of several factors and are beyond the scope of this work.

#### Production of value-added compounds

*Synechocystis *sp. PCC6803 is considered as a candidate photobiological production platform - it can potentially produce molecules of interest by using CO_2 _and light [6]. To this end, *i*Syn669 can be used to perform simulations, not only for assessing the feasibility of producing a given compound, but also to identify potential metabolic engineering targets towards improved productivity. For example, FBA simulations can help in estimating maximum theoretical yields for the products/intermediates of interest. A product of obvious interest is hydrogen. In our previous work [9], we have estimated maximum theoretical hydrogen production values that are far from the current state of experimental reports. *In silico *studies can direct the efforts and counsel the scientists towards a hydrogen producing cyanobacteria that could be of impact. *i*Syn669 predicts, in autotrophic conditions, a theoretical H_2 _evolution rate of 0.17 mmol g_DW_^-1 ^h^-1 ^obliterating biomass growth. Else, the stoichiometry permits the evolution of 0.156 mmol g_DW_^-1 ^h^-1 ^of hydrogen with a biomass growth of 10% of the wild type (0.007 mmol g_DW_^-1 ^h^-1^).

Succinate is an important metabolite for its biotechnological applications as well as for being a metabolite that bridges the TCA cycle with the electron transfer chain. As an example of the usefulness of the present metabolic model we have designed an *in silico *metabolic engineering strategy to improve the production of succinate. The underlying idea is to design a succinate over-producing metabolic network (through reaction knock-out simulations), whereas the intracellular fluxes are distributed so as to maximize the biological objective function (e.g. growth) [47]. To this end, OptGene algorithm [37] was used together with Minimization Of Metabolic Adjustment (MOMA) [17] as a biological objective function. MOMA has been reported to provide better description of flux distributions in mutants or under un-natural growth conditions as opposed to FBA. A *design objective function *which copes with the metabolite of interest, succinate, has been determined maintaining the *biological objective function *as the biomass formation.

OptGene simulations for single, double and triple knock-out strategies were performed to obtain solutions with improved succinate production, but without drastically diminishing the biomass production. We used mixotrophic conditions, for which wild type optimal growth rate was 0.17909 mmol g_DW_^-1 ^h^-1^. The best single knock-out was found to be the mutant of pyruvate kinase (reaction *2.7.1.40c *in *i*Syn669 and genes *sll0587 *and *sll1275*) that has a succinate evolution of 0.5695 mmol g_DW_^-1 ^h^-1 ^with a growth rate of 0.0714 mmol g_DW_^-1 ^h^-1^. Blocking this reaction, preventing pyruvate and phosphoenolpyruvate from using GTP and GDP would drive a high increase in succinate production. The flux between pyruvate and phosphoenolpyruvate can still be accomplished with reactions *2.7.1.40a *and *2.7.9.2*, but using ATP and ADP as cofactors. Double deletion did not improve the results from the single knock-out strain, evolving the same succinate production with the same growth rate. The best triple knock-out was found to be the combination of pyruvate kinase (reaction *2.7.1.40c *in *i*Syn669 and genes *sll0018 *and *sll0587*), fructose-bisphosphate aldolase (reaction *4.1.2.13b *in *i*Syn669 and genes *slr0943 *and *sll1275*) and succinate dehydrogenase (reaction *_1.3.99.1 *in *i*Syn669 and genes *sll0823*, *sll1625 *and *slr1233*). This simulated strain has a succinate evolution of 0.6999 mmol g_DW_^-1 ^h^-1 ^with a growth rate of 0.0688 mmol g_DW_^-1 ^h^-1^. This design combines the blocking of the oxidation of succinate on the electron chain transfer through succinate dehydrogenase with the prevention of using GTP between pyruvate and phosphoenolpyruvate and the lack of an aldolase needed in the reductive mode of the pentose phosphate pathway. This leads to a situation where flux is directed to TCA cycle in order to meet with an overproduction of succinate.

These studies on knock-outs are reaction centered, even though the *in vivo *knock-out building will ultimately be through gene manipulations. This is the reason underlying the fact that we found *2.7.1.40c *knock-out as the best result. This design would hint at the idea of selection of a mutated pyruvate kinase protein specific for ATP cofactor. This may be difficult to achieve on the bench, but has high biotechnological expectations.

### *i*Syn669 as a data integration scaffold

Apart from the flux simulations, another important problem in the field of metabolic systems biology that can be addressed by using reconstructed genome-scale models is the integration of the different genome-wide bio-molecular abundance datasets, i.e. *omics *datasets, such as transcriptome and metabolome. An example of algorithms for carrying out such an integrative analysis through the use of genome-scale metabolic networks is Reporter Features [48,49]. Reporter algorithm allows integration of *omics *data with bio-molecular interaction networks, thereby allowing identification of cellular regulatory focal points (i.e. *reporter features*), for instance *reporter metabolites *as regulatory hubs in the metabolic network.

In this work, Reporter Features software was used to integrate transcriptional information over the reconstructed *Synechocystis *sp. PCC6803 network allowing us to infer regulatory principles underlying metabolic flux changes following shifts in growth mode. In particular, we analyzed the data from a work [50] that reports the transcriptional changes caused in *Synechocystis *sp. PCC6803 by shifts from darkness to illumination conditions and back. As it can be understood from the rationale beneath the metabolic capabilities of this cyanobacterium, the presence or absence of light drives big changes in the flux distribution through the network, as discussed in the previous sections. We have focused our study on the relationship between the transcription of *Synechocystis *sp. PCC6803 genes and the reactions of the metabolic network. Associations between genes and reactions were identified, listing all the genes that performed or were involved in a specific reaction. With this information and the metabolic model, Reporter Features analysis was carried out. In brief, the analysis helped to identify metabolites around which the transcriptional changes are significantly concentrated. These metabolites are termed reporter metabolites as they represent key regulatory nodes in the network.

Gill *et al *[50] designed the experiment so that *Synechocystis *was grown to mid-exponential phase (A_730 _= 0.6 to 0.8). Then, the lights were extinguished and RNA samples were taken after 24 h in the dark (full dark). Illumination was then turned back on for 100 min (transient light), followed immediately by an additional 100 min in the dark (transient dark).

We were interested in two aspects of this study: i) to identify metabolites around which regulation is centered during the light regime transitions; and ii) to find the metabolic genes that were collectively significantly co-regulated across these transitions [49].The analysis was divided in three parts: an analysis of the data arrays from the whole experimental profile ("all time points"), an analysis of the shift from darkness to a light environment ("dark to light") and from light back to dark ("light to dark"). For a study of the overall genome and its light regulation, refer to Gill *et al *[50]. In this study, as the relationship between the metabolism and this regulation was investigated, genes with no direct relationship to a metabolic reaction were not considered. Distributions of the genes across KEGG Orthologies related to the metabolism altered with the light shift are depicted in Table 5.

**Table 5:** KEGG orthology groups for the metabolic genes altered with the light shift.

	*All time points*		*Dark to Light*		*Light to Dark*	
	
	*Number* *of genes*	*%*	*Number* *of genes*	*%*	*Number* *of genes*	*%*
Energy Metabolism	128	60.38	128	51.82	127	61.65

Amino Acid Metabolism	25	11.79	31	12.55	24	11.65

Carbohydrate Metabolism	24	11.32	28	11.33	23	11.16

Metabolism of Cofactors and Vitamins	13	6.13	26	10.53	12	5.83

Nucleotide Metabolism	12	5.66	23	9.32	12	5.83

Lipid Metabolism	7	3.3	5	2.02	6	2.91

Membrane Transport	3	1.42	4	1.63	2	0.97

Biosynthesis of Secondary Metabolites	0	0	1	0.4	0	0

Biosynthesis of Polyketides and Nonribosomal Peptides	0	0	1	0.4	0	0

Total	212	100	247	100	206	100

#### All time points

**Figure 4:**
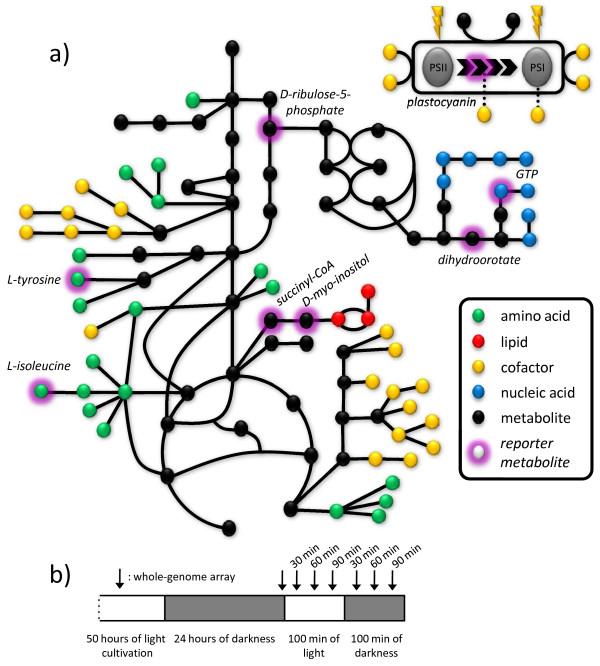
***Reporter metabolites *under light/dark regime**. a) *Reporter metabolites *for *all time points *set of arrays depicted on the *i*Syn669 network. b) Light/dark-shift profiles and localization of the genome arrays for the work from Gill *et al*. [47].

When all seven arrays were used, *reporter metabolites *were found to be quite scattered across the metabolism spanning several metabolic pathways, and thus offering a global view of the transcriptional response in the metabolic network (see Figure 4a and Table 6a). Presence of some amino acids (*L-tyrosine*, *L-isoleucine*), nucleic acids and its precursors (*GTP*, *dihydroorotate*), carbon metabolism metabolites (*D-ribulose-5-phosphate*, *succinyl-CoA*), lipids precursors (*myo-inositol*, *D-myo-inositol 3-monophosphate*), cofactors (*thioredoxin*, *p-aminobenzoate*) and photosynthesis metabolites (*plastocyanin*) pictures a scenario of a global regulation throughout the different metabolic pathways.

**Table 6:** Reporter metabolites for the light shift experiment.

a)		b)		c)	
***Metabolite***	***Number of neighbors***	***Metabolite***	***Number of neighbors***	***Metabolite***	***Number of neighbors***

**All time points**		**Dark to Light**		**Light to Dark**	

L-tyrosine	4	N-carbamoyl-L-aspartate	3	5-phosphoribosyl-N-formylglycineamidine	3

N-carbamoyl-L-aspartate	3	dihydroorotate	3	diphosphate	76

dTDP	4	5-phosphoribosyl 1-pirophosphate	9	a 1,4-alpha-D-glucan_n	2

L-isoleucine	3	L-valine	3	a 1,4-alpha-D-glucan_n1	2

D-ribulose-5-phosphate	4	5-phospho-ribosyl-glycineamide	3	UDP-N-acetylmuramoyl-L-alanyl-D-glutamyl-meso-2,6-diaminoheptanedioate	2

D-myo-inositol (3)-monophosphate	2	O-phospho-L-homoserine	2	pyridoxine-5'-phosphate	2

myo-inositol	2	peptidylproline (omega = 180)	4	(E, E)-farnesyl diphosphate	3

L-valine	3	peptidylproline (omega = 0)	4	GMP	6

succinyl-CoA	3	indole-3-glycerol-phosphate	2	phosphoribosylformiminoAICAR-phosphate	2

adenosine	2	5-aminoimidazole ribonucleotide	3	L-aspartyl-4-phosphate	2

GTP	13	tetrahydrofolate cofactors	8	pantothenate	2

thioredoxin	11	GTP	13	undecaprenyl-diphospho-N-acetylmuramoyl-L-alanyl-D-glutamyl-meso-2,6-diaminopimeloyl-D-alanyl-D-alanine	2

thioredoxin disulfide	11	L-glutamate gamma-semialdehyde	2	MurAc(oyl-L-Ala-D-gamma-Glu-L-Lys-D-Ala-D-Ala)-diphospho-undecaprenol	2

p-aminobenzoate	2	inosine-5'-phosphate	5	undecaprenyl-diphospho-N-acetylmuramoyl-L-alanyl-D-glutamyl-L-lysyl-D-alanyl-D-alanine	2

acetylphosphate	2	pantetheine 4'-phosphate	2	L-aspartate-semialdehyde	2

glycine	7	UDP-N-acetylmuramoyl-L-alanyl-D-glutamate	2	5-phospho-ribosyl-glycineamide	3

succinate	7	phytoene	2	5'-phosphoribosyl-N-formylglycineamide	4

dihydroorotate	3	thioredoxin	11	sulfur	2

PC	12	thioredoxin disulfide	11	glycine	7

*Reporter metabolites *for each set of arrays analysed with Reporter Features software.

By using the metabolic sub-network search algorithm, we found 212 genes that have their expression changed across the arrays and that have a relationship with the metabolites of *i*Syn669 network. Furthermore, 50 genes were identified that are strongly co-regulated all along the profile of the experiment (Additional File 7, section a). This set of genes is characterized in two groups. The first set consists of the genes from photosynthesis (93.85%) and oxidative phosphorylation (6.15%). The second set is representative of a variety of genes from different pathways such as amino acid metabolism (39%), carbohydrate metabolism (22%), nucleotide metabolism (13%), nitrogen metabolism (13%) and metabolism of cofactors (9%) that globally regulates the entire metabolic network (see Table 5 for further details).

It can be expected that an experimental design like the one we have based our work on, which combines a shift from dark to light with a shift back to darkness, will encompass an important part of the regulatory changes the cell is undergoing in its natural habitat. In a glucose-deficient environment, the presence or absence of light is the main condition around which the *Synechocystis *metabolism gravitates [9]. Indeed, one of the co-regulated sets consists of the genes coding for the proteins that work on, and around, the thylakoid membrane, let it be photosynthesis or oxidative phosphorylation genes.

#### Dark to light

Next, we considered the arrays that represent the shift from darkness to light, the first three arrays (from "24 hours of darkness" array to "60 minutes of light" array). *Reporter metabolites *were found to be largely within the nucleotide and amino acid metabolism (Table 6b). Some cofactors were also identified as regulation hubs like *tetrahydrofolate*, *thioredoxin *and *adenosylcobinamide*.

Sub-network search yielded set of 247 genes that have their expression changed across the first three arrays and that are related with *i*Syn669 reactions. Furthermore, 84 genes were identified that are strongly co-regulated across the three arrays (Additional File 7, section b). This set of genes cover photosynthesis (25%), oxidative phosphorylation (24%), amino acid metabolism (11%), carbohydrate metabolism (11%), nucleotide metabolism (10%) and metabolism of cofactors (10%).

This set of data arrays are indeed a good example of a cell's metabolic machinery starting up. After a 24 hour period in darkness where cell density did not change (see Figure 1 in Gill *et al *[50]), light enters the system and the cell starts to synthesize new bio-molecules, mostly nucleotides so it can copy its genetic material and amino acids to build up proteins.

#### Light to dark

Finally, we considered the arrays that represent the shift from light to dark, data from "90 minutes of light" array to "60 minutes of dark" array. Similar to the previous case study, reporter metabolites were found to be focused on the nucleotide and amino acid metabolism (Table 6c). Additionally, the presence of metabolite *a 1,4-alpha-D-glucan_n *and its cognate *a 1,4-alpha-D-glucan_n1 *also stands out as they are involved in carbon reserves catabolism and anabolism.

With the help of the sub-network search, 133 genes were identified as being significantly co-regulated across those three arrays (Additional File 7, section c). This set comprises of the genes from photosynthesis (34%), oxidative phosphorylation (26%), amino acid metabolism (12%), carbohydrate metabolism (12%), nucleotide metabolism (7.5%) and metabolism of cofactors (4.5%).

This last set of data array is a scenario where metabolism is being shut down, as a consequence of the darkness and lack of carbohydrate source. Without light, photosynthesis is blocked and carbon fixation is nearly obliterated. Cells strive to build up carbon reserves (hence the presence of *a 1,4-alpha-D-glucan_n *as a *reporter metabolite*) and oxidative phosphorylation is the main energy pathway that remains present. Regulation is centered on the energy metabolism shift (60% of the total co-regulated sub-network), withholding amino acids and nucleotide precursors and keeping the cofactors available in a low-profile metabolism.

## Conclusions

We have successfully reconstructed a genome-scale metabolic network for *Synechocystis *sp. PCC6803, called *i*Syn669, which allows simulating production of all the metabolic precursors of the organism. The metabolic reconstruction represents an up-to-date database that encompasses all knowledge available in public databases, scientific publications and textbooks on the metabolism of this cyanobacterium.

From the annotation publicly available, our metabolic network includes 882 metabolic reactions and 790 metabolites, as well as the information from 669 genes that have some relationship with the metabolic reactions. This model is the most complete and comprehensive work for *Synechocystis *sp. PCC6803 to date, which has its potential as the photosynthetic model organism. Interestingly, the reconstruction identified 79 reactions that should be present in the metabolism but with no cognate gene discovered yet; this should direct experimental work at the discovery of these genes. Topological characteristics of the network resemble those of other reconstructed microbial metabolic networks and thus provide an additional input for the analysis of their structural and organizational properties from evolutionary perspective.

Applicability of *i*Syn669 metabolic model was demonstrated by using a variety of computational analyses. Flux balance analysis was applied in order to simulate the three physiologically important growth conditions of cyanobacteria, *viz*., heterotrophic, mixotrophic and autotrophic. Our metabolic model was capable of simulating the production of the monomers or building blocks that build up the cells, in the range that is in agreement with the reported growth experiments. Our photosynthetic metabolic model includes all of the central metabolic pathways that previous works [23-25] considered. Regarding the parts from our model that overlap with the previous works (part of the central carbon metabolism), the predictions for the flux directionality changes following light shift match between those models and *i*Syn669. In fact, *i*Syn669 expands the flux study to all the pathways described in the *Synechocystis *sp. PCC6803 genome annotation. Further work should be directed at the definition of a detailed and descriptive biomass cell composition, so as to have a better representation of the biomass equation for simulation purposes.

Single reaction/gene knock-out simulations revealed 311 genes that are essential for the survival. Bearing in mind the distance from the efforts taken in the annotation of the genome of the bacteria and yeast models to that of the cyanobacterium, our study shows that *Synechocystis *sp. PCC6803 has a larger fraction of genes that are essential for producing biomass, as opposed to *Escherichia coli *and *Saccharomyces cerevisiae*. Further investigation of the causes for this difference will be of definite interest in understanding the genome annotation and/or the evolution of the metabolic network of *Synechocystis*.

Evaluation of the theoretical potential of this organism to produce hydrogen was assessed, in support of the efforts directed to this direction from several groups and scientific council initiatives. Present hydrogen production projects are far from the theoretical potential, but efforts in this field can trigger a very significant increase of the present hydrogen evolution rates in *Synechocystis *sp. PCC6803 or other photobiological production platforms candidates, e.g. *Chlamydomonas reinhardtii*, *Nostoc punctiforme *and *Synechococcus *species.

Suitability of the presented model for performing *in silico *metabolic engineering analysis was demonstrated by using OptGene software framework. Furthermore, we also show that *i*Syn669 can be used as a scaffold to integrate network-wide *omics *data. As a case study, we identified key *reporter metabolites *around which regulation during light shifts is organized, as well as gene sub-networks that were co-regulated across the light conditions.

Altogether, the genome-scale metabolic network of *Synechocystis *sp. PCC6803 (*i*Syn669) will be a valuable tool for the applied and fundamental research of *Synechocystis *sp. PCC6803, as well as for the broad field of metabolic systems biology. *i*Syn669 represents an important step for the integration of tools and knowledge from different disciplines towards development of photo-biological cell factories.

## Methods

### Metabolic network reconstruction

Pathway Tools software [33] was used to construct a *Synechocystis*-specific database of genes, proteins, enzymes and metabolites. *Synechocystis *sp. PCC6803 genome and annotation files were downloaded from NCBI Entrez Genome repository as of date 10 of September of 2008 [51]. Pathway tools retrieved a first version of the network, which had to be checked with different kinds of databases depending on the information they bear. Databases used towards this purpose included Enzyme nomenclature database [34], KEGG pathway database [27], BioCyc genome database [26], BRENDA Enzyme database [28] and UniProt protein database [29].

Parts that characterize *Synechocystis *network, like the incomplete TCA cycle [52,53], the presence of the glyoxylate shunt [35], the interconnected photosynthesis and oxidative phosphorylation [54] or the cyclic and non-cyclic electron transport related to these latter processes [55-57], were accounted for in detail.

At the end of the reconstruction process, four kinds of relationships were present in the database: reaction with cognate genes, reactions that needed to be included in the model in order to have metabolic precursors in the network (with no assigned genes), non-enzymatic reactions that have no related gene, and genes described in the annotations but with no assigned function. For an overview of the underlying process, please refer to Fortser *et al *[32] work on the reconstruction of *Saccharomyces cerevisiae *metabolic network.

### Linear programming for Flux Balance Analysis

The set of biochemical reactions of the genome-scale metabolic model were formulated as a steady state stoichiometric model:

S⋅v=0

The details are described elsewhere, for example in Stephanopoulos *et al *[40]. This model describes cellular behavior under pseudo steady-state conditions, where *S *is stoichiometric matrix that contains the stoichiometric coefficients corresponding to all internal (balanced) metabolites. *v *is flux vector that corresponds to the columns of *S*. Given a set of experimentally-driven constraints, former equation was solved by using linear programming, the approach known as flux balance analysis, or FBA [16].

Since the number of reactions is typically larger than the number of metabolites, the system becomes underdetermined. In order to obtain a feasible solution for the intracellular fluxes, an optimization criterion on metabolic balances has to be imposed. This can be formulated by maximizing one of the biochemical reactions, e.g. biomass equation, subject to the mass balance and the capacity constraints.

For instance,

$$\begin{array}{c} \text{Max}\left(\nu_{i}\right)\;\;\;\text{subject to }S\;\cdot \;\nu_{j}=\;0\;\forall j\in \text{N}\\ \nu_{j,irr}\in {\text{R}}^{+}\\ \nu_{j,rev}\in \text{R}\\ \nu_{j,const}\in \text{R},v_{min}<\nu_{j,const}<v_{max}\\ \nu_{j,\;uptake}\in \text{R},v_{min}<\nu_{j,uptake}<v_{max} \end{array}$$

where *v_j _*is the rate of the *j^th ^*reaction. The elements of the flux vector *v *were constrained for the definition of reversible and irreversible reactions, *v_j, rev _*and *v_j, irr_*, respectively. Additionally, two set of equations were established, *ν_j, const_*, constrained metabolic reactions, and *ν_j, uptake_*, uptake reactions, which were bound by experimentally determined values from the literature. Biomass synthesis was considered as a drain of precursors or building blocks into a hypothetical biomass component. Flux through biomass synthesis reaction, being the biomass formation rate, is directly related to growth of the modeled organism [40]. Table 3 shows the biomass composition that was considered in the *i*Syn669 metabolic model.

Simulations were performed with the OptGene software [37]. Some capacity constraints had to be added in order to have a feasible solution for the linear programming problem. As an example, maximum uptake rates were determined as follows: maximum glucose uptake rate under heterotrophic conditions was found to be 0.85 mmol glucose g_DW_^-1 ^h^-1 ^[23]. Maximum CO_2 _uptake rate was found to be 3.7 mmol CO_2 _g_DW_^-1 ^h^-1 ^[24]. Additionally, we fixed the maintenance requirement for the heterotrophic case to be 1.67 ATP moles per mole of glucose consumed as was determined by ref [24], and was maintained for autotrophic and mixotrophic growth.

### MOMA algorithm

Segre *et al *[17] introduced the method of minimization of metabolic adjustment (MOMA) to better understand the flux states of mutants. MOMA is based on the same stoichiometric constraints as FBA, but relaxes the assumption of optimal growth flux for the mutants, testing the hypothesis that the corresponding flux distribution is better approximated by the flux minimal response to the perturbation than by the optimal one.

MOMA algorithm searches for a point in the feasible space of the solutions space of the knock-out (Φ^j^) that has minimal distance from a given flux vector *w*. The goal is to find the vector *x *∈Φ^j ^such that the Euclidean distance

$$D(w,x)=\sqrt{\sum_{i=1}^{N}{\left(w_{i}-x_{i}\right)}^{2}}$$

is minimized. For details, please address to Segre *et al *[17].

### Reporter Features algorithm

Reporter Features software [48] works on three kinds of information - network, *omics *data and association between genes and the nodes in the network. We have used Reporter Features for a transcriptomic analysis, so our three files were *p-values *file, resulting from a Student t-test run on transcriptomic data, interaction file, where reactions are connected to the corresponding substrates and products, and association file, where gene are associated to reactions they are involved in, either by coding for the enzyme or by regulating the gene that codes for the enzyme.

In brief, Reporter algorithm converts the p-value for a given node to a z-score by using the inverse normal cumulative distribution function (cdf^-1^).

$${\text{z}}_{\text{gene i}}={\text{cdf}}^{-\text{1}}\left(\text{1}–{\text{p}}_{\text{gene i}}\right)$$

After scoring each non-feature node in this fashion, we need to calculate the score of each feature *j*, z_feature j_. We used the scoring method based on distribution of the means, which is a test for the null hypothesis "genes adjacent to feature *j *display their normalized average response by chance". In particular, the score of each feature *j *is defined as the average of the scores of its neighbour *N_j _*nodes (genes), i.e.:

$$z_{feature\;j}=\frac{1}{N_{j}}\sum_{k=1}^{N_{j}}z_{gene\;k}$$

To evaluate the significance of each z_feature *j*_, this value should be corrected for the background distribution of z scores in the data, by subtracting the mean (*m_N_*) and dividing by the standard deviation (*s_N_*) of random aggregates of size *N*.

$$z_{feature\;j}^{corrected}=\frac{\left(z_{feature\;j}-m_{N}\right)}{s_{N}}$$

## Abbreviations

BM: biomass; DCW: dry cell weight; FBA: flux balance analysis; MCA: metabolic control analysis; MOMA: minimization of metabolic adjustments; ORF: Open Reading Frame; PEP: phosphoenolpyruvate; ROOM: regulatory on-off minimization of metabolic fluxes; RuBisCO: Ribulose-1,5-bisphosphate carboxylase oxygenase; TCA cycle: tricarboxylic acid cycle

## Authors' contributions

AM and EN conducted the reconstruction and the different analyses. PF and JFU conceived of the study and participated in its design. AM and KRP designed the study and wrote the manuscript. All authors contributed to, read and approved the final manuscript.

## Supplementary Material

Additional file  1 ***i*Syn669 reactions to gene connections**. Excel file with the list of ***i***Syn669 reactions and its cognate list of genes. Click here for file

Additional file  2 ***i*Syn669 genome-scale metabolic model in OptGene format**. Text file with the stoichiometric model, in OptGene [37] format, with all the constraints needed for its simulation with FBA algorithm. Click here for file

Additional file  3 **Most connected metabolites with filtered cofactors**. Supplementary table with most connected metabolites once the cofactors have been filtered. Click here for file

Additional file  4 ***i*Syn669 metabolic fluxes simulated under four conditions**. Excel file with all the reactions simulations and resulting flux ranges from the model simulated under four growth conditions: autotrophy, dark o pure heterotrophy, light-activated heterotrophy and mixotrophy. Click here for file

Additional file  5 **Fluxes of reactions around pyruvate**. Flux values (in mmol/g DCW/h) for reactions that produce or drain pyruvate in *Synechocystis *sp. PCC6803 metabolism. Negative sign in bidirectional reactions means pyruvate consumption. Reactions names can be traced in reaction list in Additional files 2 and fluxes can be found in Additional file 4. Click here for file

Additional file 6 **FBA and MOMA simulation values for biomass growth in *Synechocystis *sp. PCC6803, *Escherichia coli *and *Saccharomyces cerevisiae *genome-scale metabolic models**. Excel file with the growth values under MOMA simulation for *Synechocystis *sp. PCC6803, *Escherichia coli *and *Saccharomyces cerevisiae*. Data for *Synechocystis *is original from present work, data for *Escherichia coli *has been obtained from metabolic model from reference 18 and data for *Saccharomyces cerevisiae *is from reference 30. Click here for file

Additional file  7 ***i*Syn669 groups of correlated genes in the three sets of arrays of light shift experiments**. Word file with the list of ***i***Syn669 correlated genes in "All time points", "Dark to light" and "Light to dark" analyses. Click here for file
