# Supplementary material for: Reconstruction and analysis of genome-scale metabolic model of a photosynthetic bacterium
Source: BMC Syst Biol. 2010 Nov 17;4:156. doi: 10.1186/1752-0509-4-156 (PMC3009638; doi:10.1186/1752-0509-4-156)
Supplement: Additional file 3 — Most connected metabolites with filtered cofactors. Supplementary table with most connected metabolites once the cofactors have been filtered. [file 1752-0509-4-156-S3.DOC]

## Most connected metabolites (cofactors filtered) in *i*Syn669 metabolic network.

| *Metabolite* | *Neighbors* | *Neighbors in* E. coli | *Neighbors in yeast* |
| --- | --- | --- | --- |
| L-glutamate | 45 | 52 | 56 |
| S-adenosyl-L-methionine | 25 | 18 | 19 |
| glutathione | 25 | 17 | 10 |
| a malonyl-ACP | 23 | 15 | 10 |
| L-glutamine | 22 | 18 | 23 |
| S-adenosyl-L-homocysteine | 21 | 12 | 14 |
| cysteinylglycine | 21 | 5 | - |
| pyruvate | 20 | 61 | 20 |
| 5-oxoproline | 20 | - | - |
| acetyl-CoA | 15 | 34 | 24 |
| L-aspartate | 15 | 23 | 20 |
| tetrahydrofolate | 14 | 10 | 13 |
| 2-ketoglutarate | 13 | 27 | 29 |
| D-glyceraldehyde-3-phosphate | 12 | 14 | 13 |
| fructose-6-phosphate | 10 | 18 | 18 |
| phosphoenolpyruvate | 10 | 26 | 12 |
| L-methionine | 10 | 15 | 15 |
| L-serine | 9 | 26 | 17 |
| 5-phosphoribosyl 1-pyrophosphate | 9 | 2 | 2 |
| isopentenyl diphosphate | 9 | 6 | 4 |
