# Supplementary figures and images for: Reconstruction and analysis of genome-scale metabolic model of a photosynthetic bacterium
Source: BMC Syst Biol. 2010 Nov 17;4:156. doi: 10.1186/1752-0509-4-156 (PMC3009638; doi:10.1186/1752-0509-4-156)

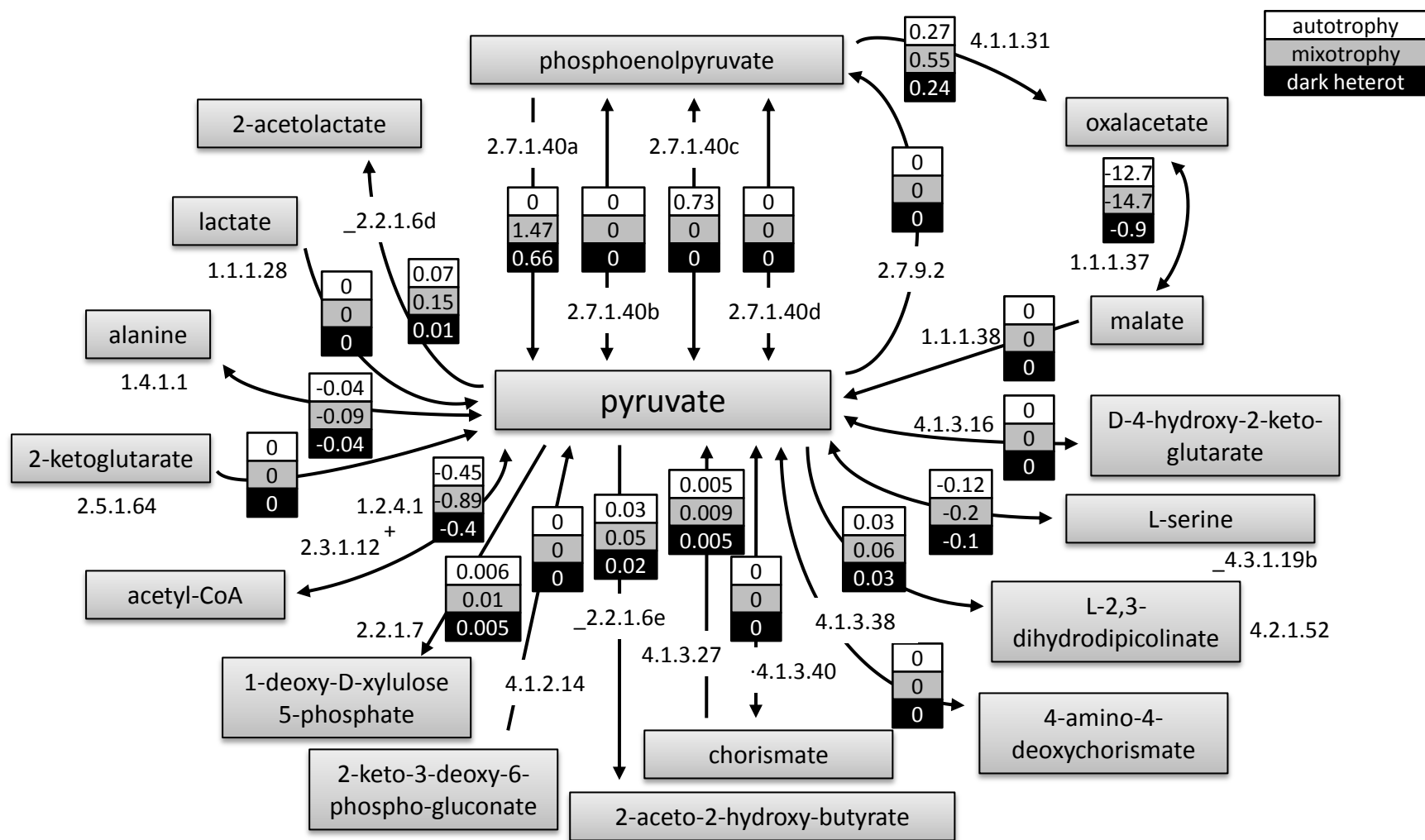

Supplement: Additional file 5 — Fluxes of reactions around pyruvate. Flux values (in mmol/g DCW/h) for reactions that produce or drain pyruvate in Synechocystis sp. PCC6803 metabolism. Negative sign in bidirectional reactions means pyruvate consumption. Reactions names can be traced in reaction list in Additional files 2 and fluxes can be found in Additional file 4. [file 1752-0509-4-156-S5.PDF]
