# Supplementary material for: Reconstruction and analysis of genome-scale metabolic model of a photosynthetic bacterium
Source: BMC Syst Biol. 2010 Nov 17;4:156. doi: 10.1186/1752-0509-4-156 (PMC3009638; doi:10.1186/1752-0509-4-156)
Supplement: Additional file 7 — iSyn669 groups of correlated genes in the three sets of arrays of light shift experiments. Word file with the list of iSyn669 correlated genes in "All time points", "Dark to light" and "Light to dark" analyses. [file 1752-0509-4-156-S7.DOC]

## Groups of correlated genes in the three sets of arrays of light shift experiments.

1. **All arrays**

- *Group I*

sll0629 (*psaK, psaK2*), sll1182 (*petC3*), sll1316 (*petC, petC1*), sll1317 (*petA*), sll1382 (*petF, fdx*), sll1796 (*petJ*), sll1899 (*ctaB*), slr0150 (*petF, fdx*), slr0342 (*petB*), slr0343 (*petD*), slr1136 (*coxB*), slr1137 (*coxA*), slr1138 (*ctaEI, ctaE, coxC*), slr1643 (*petH*), slr1655 (*psaL*), slr1828 (*petF, fdx*), slr1834 (*psaA*), slr1835 (*psaB*), sml0004 (*petN, ycf6*), sml0008 (*psaJ*), smr0003 (*petM, ycf31*), smr0004 (*psaI*), smr0005 (*psaM*), smr0010 (*petG*), ssl0020 (*petF, fed1*), ssl0563 (*psaC*), ssr0390 (*psaK, psaK1*), ssr2831 (*psaE*)

- *Group II*

sll0036, sll0250 (*dfp*), sll0593 (*glk*), sll0920 (*ppc*), sll1043 (*pnp*), sll1454 (*narB*), slr0288 (*glnN*), slr0329 (*xylR*), slr0379 (*tmk*), slr0500 (*hisB*), slr0546 (*trpC*), slr0657 (*lysC*), slr0752 (*eno*), slr0917 (*bioF*), slr0958 (*cysS*), slr0994 (*lipB*), slr1031 (*tyrS*), slr1511 (*fabH*), slr1550 (*lysS, herC*), slr1556 (*ddh*), slr1722 (*guaB, gnaB*), slr1756 (*glnA*), slr2035 (*proB*), slr2072 (*ilvA*), ssr1720 (*tyrS*)

1. **Dark to light**

sll0036, sll0223 (*ndhB*), sll0373 (*proA*), sll0450 (*norB*), sll0461 (*proA*), sll0519 (*ndhA*), sll0520 (*ndhI*), sll0521 (*ndhG*), sll0629 (*psaK, psaK2*), sll0646 (*cya2*), sll0920 (*ppc*), sll0927 (*metX*), sll1161 (*cya3*), sll1182 (*petC3*), sll1220 (*hoxE*), sll1221 (*hoxF*), sll1223 (*hoxU*), sll1258 (*dcd*), sll1262 (*ndhN*), sll1316 (*petC, petC1*), sll1317 (*petA*), sll1329, sll1343 (*ape2, pepN*), sll1370 (*rfbM*), sll1383 (*suhB, ssyA*), sll1415, sll1732 (*ndhF3*), sll1796 (*petJ*), sll1899 (*ctaB*), slr0090 (*ppd*), slr0212 (*metH*), slr0261 (*ndhH*), slr0331 (*ndhD1*), slr0342 (*petB*), slr0343 (*petD*), slr0348, slr0400, slr0493 (*rfbM*), slr0597 (*purH*), slr0657 (*lysC*), slr0697, slr0710 (*gdhA*), slr0752 (*eno*), slr0787, slr0839 (*hemH, scpA*), slr0844 (*ndhF1*), slr0897, slr0898 (*nirA*), slr0917 (*bioF*), slr1030 (*chlI*), slr1055 (*chlH*), slr1136 (*coxB*), slr1137 (*coxA*), slr1138 (*ctaEI, ctaE, coxC*), slr1159 (*purD*), slr1279 (*ndhC*), slr1280 (*ndhK, psbG1*), slr1281 (*ndhJ*), slr1291 (*ndhD2*), slr1351 (*murF, mra*), slr1623 (*ndhM*), slr1655 (*psaL*), slr1722 (*guaB, gnaB*), slr1777 (*chlD*), slr1791 (*cysH*), slr1834 (*psaA*), slr1835 (*psaB*), slr1874 (*ddlA*), slr1991 (*cya1*), slr2007 (*ndhD5*), slr2009 (*ndhD6, ndhF*), slr2074 (*manA, pmi*), sml0004 (*petN,* *ycf6*), sml0008 (*psaJ*), smr0003 (*petM, ycf31*), smr0004 (*psaI*), smr0005 (*psaM*), smr0010 (*petG*), ssl0563 (*psaC*), ssr0390 (*psaK, psaK1*), ssr1386 (*ndhL, ictA*), ssr2831 (*psaE*)

1. **Light to dark**

sll0045 (*spsA, sps*), sll0135, sll0223 (*ndhB*), sll0258 (*psbV*), sll0362 (*alaS, lovB*), sll0373 (*proA*), sll0421 (*purB*), sll0422, sll0427 (*psbO*), sll0461 (*proA*), sll0469 (*prsA, prs*), sll0519 (*ndhA*), sll0520 (*ndhI*), sll0521 (*ndhG*), sll0585, sll0593 (*glk*), sll0629 (*psaK, psaK2*), sll0660 (*pdxA*), sll0753 (*folD*), sll0823 (*sdhB*), sll0851 (*psbC*), sll0868 (*lipA, lip*), sll0900 (*hisG*), sll0920 (*ppc*), sll1027 (*gltD*), sll1043 (*pnp*), sll1056 (*purL, purI*), sll1182 (*petC3*), sll1184 (*ho1*), sll1194 (*psbU*), sll1220 (*hoxE*), sll1221 (*hoxF*), sll1223 (*hoxU*), sll1262 (*ndhN*), sll1281 (*psbZ, ycf9*), sll1316 (*petC, petC1*), sll1317 (*petA*), sll1322 (*atpI, atpB*), sll1323 (*atpG*), sll1324 (*atpF*), sll1325 (*atpD, atpH*), sll1326 (*atpA*), sll1327 (*atpC, atpG*), sll1370 (*rfbM*), sll1382 (*petF, fdx*), sll1418 (*psbP, psbP2*), sll1435 (*pet112*), sll1459, sll1625 (*sdhB*), sll1732 (*ndhF3*), sll1796 (*petJ*), sll1815 (*adk*), sll1867 (*psbA3, psba-3*), sll1875 (*ho2*), sll1899 (*ctaB*), slr0014 (*mgtC*), slr0033, slr0090 (*ppd*), slr0150 (*petF, fdx*), slr0261 (*ndhH*), slr0329 (*xylR*), slr0331 (*ndhD1*), slr0342 (*petB*), slr0343 (*petD*), slr0357 (*hisS, hisS1*), slr0370 (*gabD*), slr0493 (*rfbM*), slr0519, slr0649 (*metS*), slr0661 (*proC*), slr0738 (*trpE*), slr0838 (*purM, purG*), slr0844 (*ndhF1*), slr0851 (*ndbA, ndh*), slr0877, slr0927 (*psbD2*), slr1136 (*coxB*), slr1137 (*coxA*), slr1138 (*ctaEI, ctaE, coxC*), slr1159 (*purD*), slr1181 (*psbA1, psba-1*), slr1233 (*frdA*), slr1279 (*ndhC*), slr1280 (*ndhK, psbG1*), slr1281 (*ndhJ*), slr1289 (*icd*), slr1291 (*ndhD2*), slr1311 (*psbA2, psba-2*), slr1325 (*spoT*), slr1329 (*atpB, atpD*), slr1330 (*atpE, atpC*), slr1418 (*pyrD*), slr1542, slr1559 (*aroE*), slr1598 (*lipA, lip*), slr1623 (*ndhM*), slr1643 (*petH*), slr1645 (*psb27*), slr1655 (*psaL*), slr1743 (*ndbB, ndh*), slr1784 (*bvdR*), slr1791 (*cysH*), slr1793 (*talB, tal*), slr1828 (*petF, fdx*), slr1834 (*psaA*), slr1835 (*psaB*), slr1979 (*trpE*), slr2007 (*ndhD5*), slr2009 (*ndhD6, ndhF*), slr2074 (*manA, pmi*), slr2081 (*tyrA*), sml0001 (*psbI*), sml0002 (*psbX*), sml0003 (*psbM*), sml0004 (*petN, ycf6*), sml0005 (*psbK*), sml0008 (*psaJ*), smr0001 (*psbT, psbTc, ycf8*), smr0003 (*petM, ycf31*), smr0004 (*psaI*), smr0005 (*psaM*), smr0006 (*psbF*), smr0007 (*psbL*), smr0008 (*psbJ*), smr0010 (*petG*), ssl0020 (*petF, fed1*), ssl0563 (*psaC*), ssl2598 (*psbH*), ssl2615 (*atpH, atpE*), ssr0390 (*psaK, psaK1*), ssr1386 (*ndhL, ictA*), ssr2831 (*psaE*), ssr3451 (*psbE*)
